# Dissociating Variability and Effort as Determinants of Coordination

**DOI:** 10.1371/journal.pcbi.1000345

**Published:** 2009-04-10

**Authors:** Ian O'Sullivan, Etienne Burdet, Jörn Diedrichsen

**Affiliations:** 1 Wolfson Centre for Cognitive Neuroscience, School of Psychology, University of Wales, Bangor, United Kingdom; 2 Department of Bioengineering, Imperial College London, London, United Kingdom; University College London, United Kingdom

## Abstract

When coordinating movements, the nervous system often has to decide how to distribute work across a number of redundant effectors. Here, we show that humans solve this problem by trying to minimize both the variability of motor output and the effort involved. In previous studies that investigated the temporal shape of movements, these two selective pressures, despite having very different theoretical implications, could not be distinguished; because noise in the motor system increases with the motor commands, minimization of effort or variability leads to very similar predictions. When multiple effectors with different noise and effort characteristics have to be combined, however, these two cost terms can be dissociated. Here, we measure the importance of variability and effort in coordination by studying how humans share force production between two fingers. To capture variability, we identified the coefficient of variation of the index and little fingers. For effort, we used the sum of squared forces and the sum of squared forces normalized by the maximum strength of each effector. These terms were then used to predict the optimal force distribution for a task in which participants had to produce a target total force of 4–16 N, by pressing onto two isometric transducers using different combinations of fingers. By comparing the predicted distribution across fingers to the actual distribution chosen by participants, we were able to estimate the relative importance of variability and effort of 1∶7, with the unnormalized effort being most important. Our results indicate that the nervous system uses multi-effector redundancy to minimize both the variability of the produced output and effort, although effort costs clearly outweighed variability costs.

## Author Summary

When performing actions, we often have many options of how to combine muscles or limbs to achieve a desired outcome. Despite this freedom, certain effector combinations are used consistently. Why? To examine this question, we asked participants to press one button with a finger from the left hand and another button with a finger from the right hand simultaneously. Participants' goal was to generate a combined force that would match a target force as accurately as possible. Different fingers have distinct strength and noise characteristics, allowing us to examine if participants combined the two fingers to minimize the overall effort or the overall variability. Prior studies have been unable to dissociate these two factors, because they have focused on movements without multi-effector redundancy, where noise always increases with increasing levels of force. With our simple protocol, we show that subjects coordinate the two fingers to minimize mainly effort, but also variability, in a proportion of 7∶1. This result suggests that the nervous system learns to coordinate different muscles or limbs by considering both effort and noise information simultaneously. These results have important implications for understanding how the brain relearns to coordinate movements following injury or stroke.

## Introduction

The motor system is highly redundant: the same task can always be accomplished by many different sequences of motor commands [1]. Part of this redundancy is caused by the fact that there are often multiple muscles or effectors that can produce the same desired effect. Thus, in the case of multi-effector redundancy, the brain has to choose how to distribute a given task across the set of muscles.

Despite the infinite number of possibilities, the motor system appears to prefer particular solutions. For example, when moving the wrist, we combine the action of different forearm muscles in a predictable, cosine-tuning-like fashion [2]. To explain these regularities, we can ask why the brain is coordinating movements this way [3], i.e. we can propose a hypothetical cost function that the biological system minimized over the course of learning. By determining the form of this cost function, and by assuming that the nervous system had sufficient exploration of the task dynamics to find an optimal solution, we can make testable predictions about how biological movements should be produced under a given task constraint.

A number of different cost functions for biological movements have been proposed [4]–[6]. Most of these studies have addressed movements for which the redundancy is temporal: here there may be only one muscle with the desired effect, but there are still many different ways of distributing the motor commands over the movement period. For example, of all the possible shapes of arm or eye movement, the motor system consistently chooses a bell-shaped velocity profile [7].

Different components of cost functions can generally be divided into two classes: effort and variability costs. Effort costs usually take a form of the sum of the squared muscle activations or motor commands [8],[9]. Alternatively, both Harris and Wolpert [5] and Burdet and Milner [10] proposed that the nervous system chooses the sequence of motor commands that minimizes the variability at the endpoint of a movement. Under the assumption of signal-dependent noise, i.e. noise that increases monotonically with the motor command, this model can predict important characteristics of the control of both arm and eye.

While effort and variability costs have different theoretical implications for the learning mechanism that is involved in the optimization of motor behaviours, they make very similar predictions concerning the temporal shape of the optimal movement. Indeed, it can be shown that the requirement to reduce variability under signal dependent noise leads to a term in the cost function that penalizes the sum of the squared motor commands over the movement, identically to the term commonly associated with effort [11]. Thus, for motor behaviours with mainly temporal redundancy, variability and effort costs are hard to dissociate.

For motor behaviours with multi-effector redundancy, however, the minimization of variability costs and the minimization of effort costs can lead to substantially different predictions concerning the distribution of work across effectors, because the noise and effort characteristics of different effectors can be partly independent. Here we study how humans distribute work across different fingers when they have to produce a given target force. By measuring the independent noise characteristics and the maximal force of the finger, we can dissociate the influence of variability and effort costs on coordination.

## Results

### Variability costs alone do not predict force sharing

**Figure 1:**
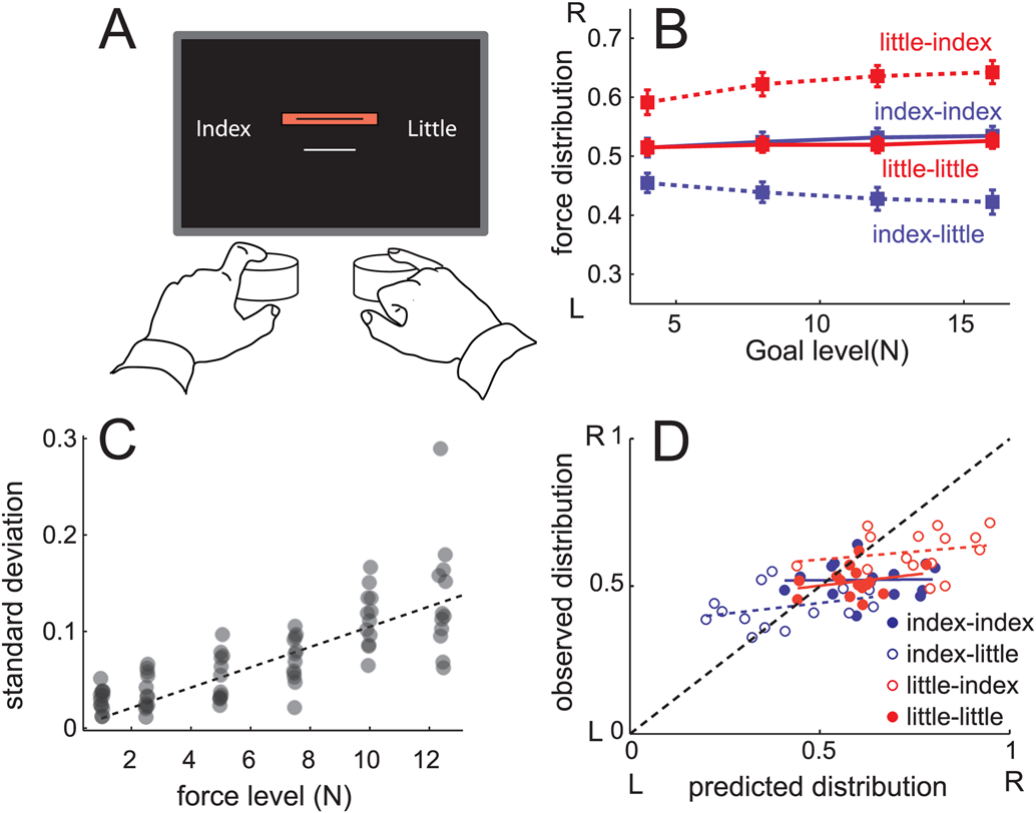
Bimanual force production task. (A) Participants pressed with a finger (index or little) of the left and the right hands on isometric force transducers. The task was to match the goal force (red line) as accurately as possible with the summed force (white line). (B) Relative distribution of forces across fingers (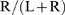) depending on goal force level and finger combination, averaged over all participants. (C) Standard deviation (N) of a representative index finger of one participant as a function of the mean produced force level. The slope of the regression line corresponds to the coefficient of variation. (D) Optimal solution for force distribution across finger based on assumption that only variability was optimized (x-axis) vs. produced force distribution (y-axis).

Fifteen neurologically healthy participants performed the simple force production task depicted in Figure 1A. The goal of the task was to produce a certain total force to match a cursor to a goal on the screen as accurately as possible by pushing onto two force transducers. Participants used 4 possible finger combinations of index and little fingers of the left and right hands. Participants had to maintain a summed force level of 4, 8, 12 or 16 N for 7 s and were given points after each trial, inversely proportional to their produced mean squared error. As a variable of main interest, we analyzed the distribution of forces (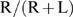) across the two fingers for the last 5 s.

Each participant produced a replicable distribution of force for each finger combination, generally producing more force with the index than with the little finger and more with the right than left hand (Figure 1B). While each chosen force distribution could be measured quite reliably (SE = 0.015), there was considerable between-person variability in the chosen solution. Furthermore, for lower force levels, participants distributed the forces more evenly across the fingers, as evidenced by the significant finger-combination x force-goal interaction, F(9,126) = 8.282, p<.001.

What cost function determines the individually chosen distribution of force? We first considered the idea that participants optimized their motor output to simply minimize the expected squared error between the sum of produced forces (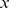) and the goal (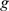). This term can be broken down into the systematic and variable error.
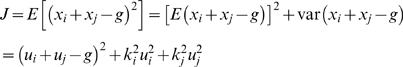
(1)


We here assumed that the force produced by each finger (*x*) is equal to the motor command (*u*) plus a noise component with a SD of 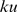. We also assumed that the noise sources for the two fingers are independent. The optimal distribution (for a full derivation see Methods) of forces for the finger combination *i, j* is
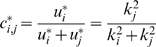
(2)


To test this model, we measured the coefficient of variation (*k*) for each of the fingers involved. We asked participants to produce a range of force levels with each finger alone and measured the SD of the force produced over a 5 s period. As shown for an exemplary finger (Figure 1C), the SD increased linearly with force, allowing us to estimate the coefficient of variation as the slope of the regression line (see Methods). Systematic deviations from linearity were observed for small forces, where the coefficient of variation was higher [12]. Overall, the coefficients of variation (k) were 1.11% (between subject SD = 0.4) and 0.82% (SD = 0.26) for left and right index fingers, and 1.61% (SD = 0.66) and 1.31% (SD = 0.46) for left and right little fingers, respectively.

Based on these measurements, we can predict the theoretically optimal distribution for each participant and finger combination and compare these to the actual distribution produced (Figure 1D). As can be seen by the deviation from the unity-line, this parameter-free model predicted a much more asymmetric force distribution than was observed. The model captures qualitatively, however, the correct difference between the different finger combinations; for example, when a little finger is combined with an index finger, the model correctly predicts a greater contribution from the index. Furthermore, regression analysis within each finger combination across participants (lines in Figure 1D) showed some relationship between the individual's ratio of noise coefficients (Eq. 2) and the individuals chosen distribution of forces, t(55) = 1.37, p = .0871. Thus, although the variability-only cost function clearly failed to predict the chosen distribution accurately, these results indicate that variability may play a role in the choice of distribution for each participant.

### Relative influence of effort and variability costs

We therefore considered a cost function that also included terms to represent effort. Effort is often conceptualized as the overall sum of the squared motor commands [6]. Inclusion of such a term would predict a symmetric distribution of forces across the fingers; when wanting to produce 10 N total, 5^2^+5^2^ is the smallest sum of squares possible. Biological systems that seek to minimize fatigue and energy expenditure, however, will likely recruit the stronger effector more. Thus, it has been suggested to normalize the motor commands by the maximum voluntary contraction (MVC) of each muscle or effector before squaring [13]. Because we have no a-priori knowledge of which effort term is appropriate, we allow here any mixture of the non-normalized effort (weighted by 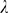), normalized effort (weighted by 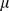), and the squared error (weighted by 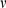):
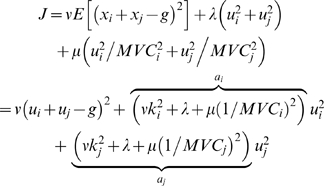
(3)


With the introduction of the collective terms 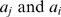, the optimal distribution can be expressed as:
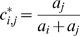
(4)


**Figure 2:**
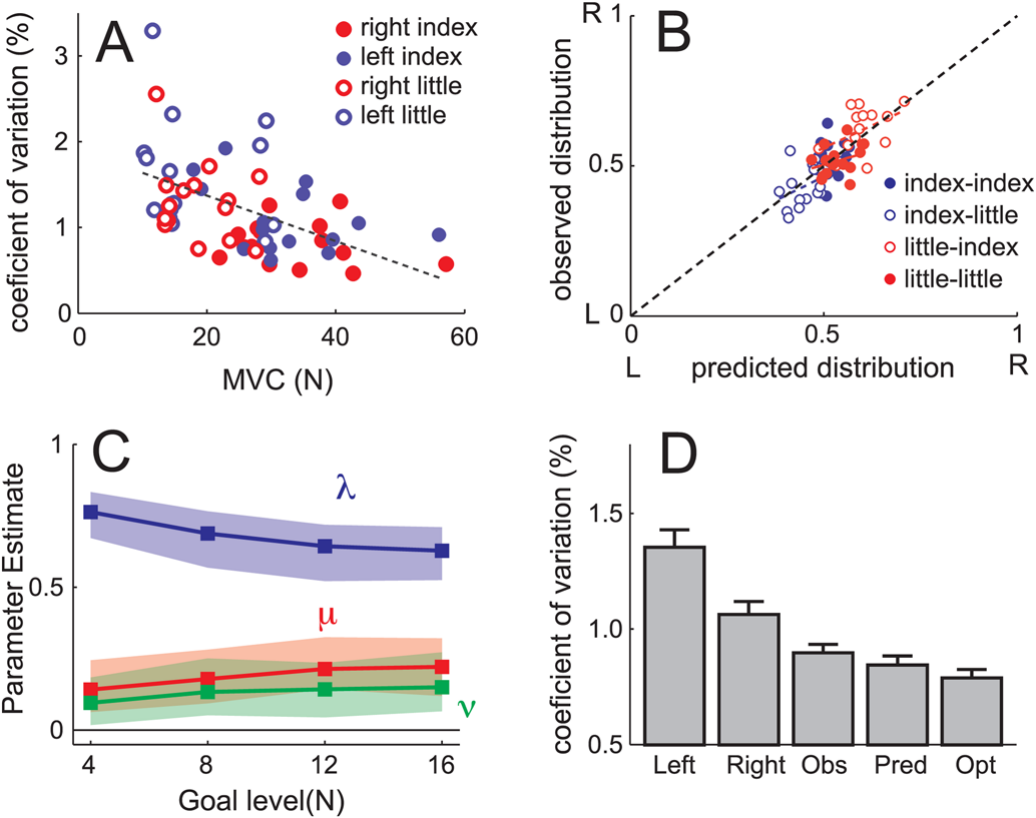
Effort and variability cost model. (A) Maximum voluntary contraction (MVC) only correlates modestly (r = −.48) with the coefficient variation of that finger across all fingers and participants. (B) Predicted force distribution following the best fitting model (x-axis) vs. observed distribution for all participants and finger combinations (y-axis). (C) Parameter estimate (+−95% confidence intervals) for the parameters for effort (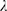), normalized effort (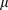), and accuracy costs (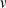) for all force levels. (D) Coefficient of variation (CV) for left: left finger alone, right: right finger alone, obs: observed in bimanual trials, pred: calculated based on unimanual CVs for the observed combination of fingers; opt: optimal CV based on unimanual CVs and optimal combination (Eq. 1).

To estimate the weight of the normalized effort cost, we also measured the MVC for each finger and participant (see Methods). The mean MVC was 34.33 N (SD = 10.50) and 36.94 N (SD = 8.72) for the left and right index, and 17.74 N (SD = 7.58) and 19.93 N (SD = 5.59) for left and right little finger respectively. Thus, the MVC showed a similar difference between fingers as the coefficient of variation 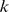. Indeed, there was a clear relationship between MVC and variability of each finger (Figure 2A, 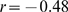). Thus, it is possible that any influence of variability onto force sharing is caused indirectly by the fact that the less noisy fingers are also stronger. We also observe, however, fingers that are relatively weak, but nonetheless able to fairly accurately produce forces over the required force range. Given this partial independence, we can ask whether the variability term will contribute to the fit over and above the two effort terms.

We therefore fit the full model to the data and used Markov Chain Monte Carlo (MCMC, see Methods) sampling to find confidence bounds of the parameter estimates (Figure 2C). To make the size of the parameters comparable - cost functions are inherently unit-less - we standardized terms to give each a prior equal weighting and constrained the parameters to sum to 1 (see Methods). Therefore, the model had 2 free parameters. For all force levels, the unnormalized effort (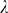) had the highest influence, whereas the two terms based on the maximal strength (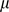) and the coefficient of variation (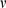) were less important. However, both latter parameters were significantly different from zero for all force levels (p<0.05). The full model predicts 55% of the remaining variance over a model that includes only 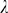 (Figure 2B).

To test whether the variability term contributed significantly to the fit, we tested the full model against a model that only included the two effort terms. To correct for the different numbers of free parameters, we used MCMC sampling to estimate the marginal likelihood of each model (see Methods). The full model provided a better explanation for the data by a Bayes factor of 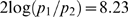 (strong evidence, [14]). All other models including any one or two of the three possible cost terms were less likely than this closest competitor (and the results were confirmed using AIC and cross-validation, see Table S1). Thus, our data clearly shows the influence of both effort and variability terms in determining the distribution of work across effectors in a redundant motor task. Variability, however, only contributed roughly 13% of the overall cost.

For the given parameter estimates of effort and accuracy costs, the model also predicts a very small systematic undershoot of the target (see Methods) of 0.065%. The mean observed undershoot was 0% in mean (SD = 0.34%), not significantly different from the prediction (p = .142). Thus, a model in which participants attempt to minimize effort and the mean-squared error can account for the data quite well.

### Feedback control and independence of noise sources

Our model relies on two simplifications. First, our model is purely feed-forward, without consideration of feedback control. To test the influence of feedback, we constructed a linear model in which the system could sense the sum of the forces of the left and the right hand (delayed by 100 ms) and change the motor commands in response. The motor commands influenced the forces via a double-exponential low pass filter [11]. When solving the cost functions (Eq. 1,3) for this system, the optimal solution comes no longer in form of optimal motor commands (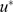), but in terms of optimal feedback gains (L). The motor commands then become a linear function of the estimated state of the system 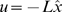 [15]. When simulating this system in presence of noise, the average produced mean force for each finger is identical to the solution of the simpler feed-forward model used here.

The second simplification is our assumption that the noise sources of the left and the right finger are independent. This assumption is relatively hard to test, as the correlation between the fingers is also influenced by the presence of feedback corrections. Indeed, for the redundant task used here, optimal feedback control predicts a negative correlation between the left and the right hand [8],[16],[17]. This is because the optimal feedback gains allow variability in the task-irrelevant dimension (the difference between the finger forces) to accumulate, while minimizing the variability in the task-relevant dimension (the sum of the finger forces). In accordance with this prediction, we found on average a within-trial correlation between the two forces of r = −.277 (between-subject SD = .092).

To determine whether this correlation was task-dependent, we used an independent bimanual force production task, in which participants had to bring separate lines, one for each finger, to two separate goals. Under a cost function that penalized the squared deviation from the right and left targets independently, the optimal feedback control law becomes independent for the two fingers [16]. Therefore the produced forces in the task should be uncorrelated. If, however, the noise of the two fingers was positively correlated, we found in simulations that the correlation of the produced forces should be correlated with roughly the same size. We then tested four participants, producing all 16 combinations of 3,4,5, or 6 N with the left and the right index fingers. In this independent force production task, we found no significant within-trial correlation between the finger forces; the observed mean correlation was r = .017 (between-subject SD = .056). These results therefore indicate that the noise source for the two fingers are close to independent and that correlation between the fingers arises from task-dependent feedback control.

### Actual performance

The large influence of effort-based cost terms on the force distribution across fingers suggests that participants settled for a solution that sacrificed accuracy. But how much better could they have performed, had they only minimized variable error?

To determine this, we calculated the theoretical optimal coefficient of variation (Figure 2D, opt), produced under a force distribution that minimizes the variable error alone (Eq. 2). For this calculation we used the measured coefficient of variation for each finger from the unimanual data and assumed equivalent performance during bimanual trials. Using the same assumptions, we also determined the predicted coefficient of variation for the actually produced force distribution (pred). This calculation indicates that the solution that participants chose should have only led to a 7.2% increase in SD compared to the lowest theoretically achievable coefficient of variation.

The actually produced coefficient of variation during bimanual trials (obs), however, was another 6.8% higher than the latter predicted value, t(59) = 2.55, p = 0.013. This indicates that there is some loss of accuracy due to simultaneous feedback control of two fingers. The produced bimanual variability, however, was 8% smaller than that produced by the better of the two fingers alone (left or right, whichever was better for a particular finger combination), t(59) = −2.49, p = 0.015. Thus, while participants did not fully achieve the predicted level of performance, our results demonstrate that sharing the force between two fingers reduces overall motor variability.

## Discussion

Our experiments show how effort and variability costs influence the way the brain distributes work across different effectors when different combinations can be used to accomplish the same task (multi-effector redundancy). It has been shown that the contribution of different arm muscles to the movement of the wrist joint can be explained using a cost function that includes the sum of squared motor commands [6]. This work, however, could not determine the source of the costs. Here, we relied on the natural differences in noise and effort characteristics across different effectors to distinguish the influence of costs arising from effort and from variability of the produced outcome. Our results demonstrate that variability significantly influences coordination. If, however, the behaviour of participants was entirely determined by the minimization of variability, participants should have combined the fingers according to the ratio of the squared coefficient of variation of the two fingers. We observed a much more even distribution across fingers, indicating a substantial influence of effort in the optimization process. Using a formal model, we were able to estimate the weight given to variability and effort separately. To our surprise variability accounted for only 13% of the total cost function, although participants were clearly rewarded based on the squared error. The increase in variability that participants took into account through the high weighting of effort, however, was relatively small. This is partly because using the stronger finger more (minimizing the normalized effort costs) also reduced the variability, as stronger finger on average also had lower coefficient of variations.

It is noteworthy that effort costs, the sum of squared motor commands, are not directly related to the minimization of energy. The energy expended by a muscle is related most closely related to the mechanical work (Nm), and under isometric condition to the sum of the produced forces over time [18]. The main justification for using the squared motor commands is that this term, but not the simple sum of the forces, predicts motor behaviour well [6]. In our task, addition of a term that penalizes the sum of motor commands, would not have influenced the predictions concerning the force distribution, as any force distribution would be equivalent.

The contrast of effort and variability costs has been extensively discussed in the literature [5],[9]. The relative importance of these two terms, however, has not been determined. This is because previous work has mostly focussed on movements, in which there are many ways to distribute motor commands across time (temporal redundancy). For this class of movements - under the assumption of signal-dependent noise - effort and variability costs predict the same or very similar temporal sequences of motor commands [11].

There is evidence, however, that a combination of variability and effort costs also determines the shape of temporally redundant movements. For example, participants increase impedance (stiffness) of the arm to compensate for unstable dynamics [19], thus indicating that variability can be minimized at the expense of effort. When dynamics become stable, impedance decreases again under the pressure of effort costs, sacrificing accuracy. A neurological disassociation between effort and variability costs has been recently observed with Parkinson's disease patients [20]. The patient group showed no losses in accuracy, when matched for movement speed with a control group. When instructed, they were able to move as fast as control participants, however, their motor system appeared to be much more reluctant to do so, indicating that effort costs were set abnormally high. These results raise the possibility that variability and effort costs may be estimated in different structures of the nervous system.

While a constant noise-to-signal ratio and quadratic cost function would predict that the distribution of force across the fingers was independent of the size of the goal, our data showed a more symmetric distribution of force across fingers for low force levels. This finding can be explained when taking into account that the SD for low force levels is higher than predicted by a simple linear relationship (Figure 1C) [12]. This means that the derivative of the SD in respect to the motor command is lower for lower force levels, such that the importance of the measured constant k in determining force sharing should be reduced. Congruent with this prediction, we found lower estimates of the parameter 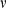 for lower force levels. Thus, our data provides evidence that participants have taken these nonlinearities into account when determining how to share forces between effectors.

In summary, our results shows that both effort and variability costs are taken into account when solving the problem of multi-effector redundancy. By exploiting the natural variability of noise and force characteristics of different fingers, we provide insight into the dissociation of two determinants of motor behaviour that so far have been closely intertwined.

## Methods

### Participants

Fifteen healthy adults (age 21, SD 4 years, 7 females) from the student population at Bangor University served as participants. Data from a 16th participant was removed, as the person had difficulties to perform the unimanual task with the little finger. We assessed handedness using 10 questions from the Edinburgh Inventory [21]. Three participants were left-handed. We therefore also analyzed the data in terms of dominant/non-dominant hand with qualitatively very similar results. Because the MVC and noise coefficients were not reversed between the left and the right side for left- compared to right-handers, we report here the results in terms of the left and right hand. Participants were recompensed with either course credit or cash payment. All experimental and informed consent procedures were approved by the Ethics committee of the School of Psychology, Bangor University.

### Apparatus

Participants sat in front of a computer monitor with forearms supported on a flat desktop. Two force transducers were placed on the table in front of the participant. The participants applied force to the cylindrical transducer (40 mm diameter, 30 mm height) with either their index or little finger. The experimenter ensured that the forearms and wrist remained on the table surface. Participants received continuous visual feedback about the total force produced through a 20-mm white horizontal cursor line, shown on an LCD display. It moved upward (230 mm = 25 N) as force was applied to the transducer, starting 25 mm from the base of the screen (no force). The target was represented on screen by a box of size 30 mm*4 mm with a black line in the middle, such that constant and variable error could be exactly determined by the participant. Force was sampled at 200 Hz.

### Bimanual trials

At the start of each trial, a message on the screen indicated the fingers to be used on this trial (Figure 1A). After participants touched both force transducers a target appeared at a height representing the required force level. The task was to apply the required force level as accurately as possible for 7 seconds, after which the target returned to the bottom of the screen. Feedback on the mean squared error, calculated over the last 6 s was given as feedback at the end of each trial. A running score for the current block of trials, with the number of points inversely related to the mean squared error, was presented throughout the experiment.

The experiment was split into 12 blocks of 48 trials run over 2 sessions separated by 1–9 days. Blocks alternated between the unimanual (see noise measurements) and bimanual conditions. In every bimanual block, all 4 possible finger combinations were tested sequentially. The sequence of combination within each block was determined pseudo-randomly. Each combination was tested 3 times with force goals set at 4, 8, 12 or 16 N; the sequence of goal levels was fully randomized.

A set of separate participants was studied in an independent force production task, in which the force for each finger was signaled with separate lines. Two goals lines were presented on each trial and participants had to match the force on each finger to the goal on that side. Every of the 16 force combinations of the levels 3,4,5 and 6 N was repeated 8 times during the experiment, only using the two index fingers. All other experimental details were the same as in the previous experiment.

### Noise measurement

In the intervening unimanual blocks, each finger was tested individually at different force levels using the same task as in bimanual trials. Index fingers were tested at 1, 2.5, 5, 7.5, 10, or 12.5 N. Due to the force limitations, for the little fingers, the 12.5 N target was not included. Each target was tested twice for each finger. The SD for the force produced was calculated over the last 5 s of each trial. We then determined the coefficient of variation using a linear regression of the SD against the mean force produced on the trial, constraining the intercept to 0. Because SD estimates have strongly skewed distributions and summary statistics are highly susceptible to outliers, we used robust regression [22] with a bisquare weighting function to determine the coefficient of variation. Correspondingly, we also used the same robust techniques to determine the mean coefficient of variation across trials for Figure 2D.

### MVC measurement

After the experiment, or in a few cases on a separate day, MVC was measured for each finger. During 3 trials of 7 s each, participants were instructed to “try to reach the highest force possible”. Visual feedback of the produced force was presented on the screen. At least 21 s elapsed between each trial to allow muscles to recover. For each trial, the mean of the highest 5% of the samples was determined, and the highest score of the three available trials was taken as the MVC measurement for the finger.

### Optimal control models

We determined the optimal motor commands (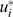) for the fingers 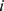, 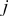 under a number of different cost functions. The force of each finger (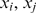) was modelled as a random variable with a mean equal to the motor command,
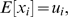
and a standard deviation proportional to the motor command, 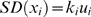, with 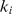 being the coefficient of variation determined by measurement for each finger. The instructed task (enforced by the point feedback on the screen) was to minimize the mean squared error between produced and required force.

Therefore, we first considered a cost function in which participants simply attempted to achieve the lowest squared error (Eq. 1). By using 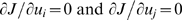 the optimal force command was determined as:
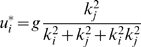
(5)from which the optimal force distribution 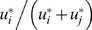 follows.

Secondly, we constructed a cost function that included both variability and effort costs. For the effort component, we chose to include both the sum of the squared motor commands (weighted by 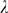), and the sum of the squared motor commands, normalized by the maximum voluntary contraction (weighted by 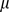) [13]. Finally, 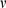 is included as a weighting factor for accuracy (Eq. 3). To make the weighting factors comparable across the 3 different terms, we added normalization factors
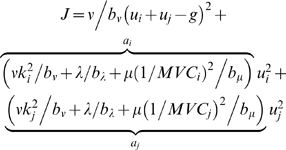
(6)with 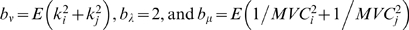. While the scaling of each component does not change the optimal solution, the scale of each parameter was adjusted, such that the numerical estimate for each parameter would be the same if the terms had equal influence on the chosen solution. Because cost functions are unit-less, and an overall scaling factor does not matter, we constrained the sum of the free parameters to be 1. The optimal force command is for finger *i* is
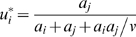
(7)with symmetrical results for finger *j*. From this one can calculate the optimal force distribution (Eq. 4) and undershoot of the target.

### Model fitting and comparison

We considered all possible cost functions arising from combinations of the non-normalized effort (weighted by 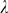), the normalized effort (weighted by 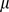), and accuracy (weighted by 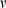). Because simple scaling of costs functions do not change the predictions, we constrained the sum of all involved weighting parameters to be 1, thereby reducing the number of free parameters. Assuming Gaussian noise with variance 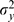, the log-likelihood of the observed force distributions 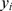, under the model 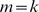, the model parameters 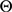 and the corresponding prediction 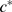 (Eq. 4) becomes
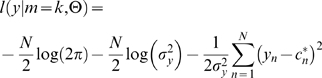
(8)


For each model we found the maximum-likelihood estimates for the free parameters 
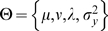
 by maximising Eq. 8 numerically. To approximate the posterior distribution of the parameters for each model 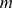 (assuming equal prior probability of all models), we drew 10000 MCMC samples of parameter values [14] using the exponential of Eq. 8 as a non-normalized posterior distribution, and discarded the first 200 samples. From the remaining samples, we calculated 95% confidence intervals of the parameters, depicted in Figure 2C. For model comparison we also estimated the marginal likelihood 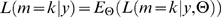, by averaging the exponential of Eq. 8 over all MCMC samples. The Bayes-factor 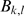 between model 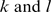 is then the likelihood ratio of the two models, allowing for a model comparison that takes into account the number of free parameters [14].
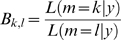
(13)


## Supporting Information

Table S1 Model comparison of cost functions
(0.06 MB DOC)
