# Supplementary material for: Dissociating Variability and Effort as Determinants of Coordination
Source: PLoS Comput Biol. 2009 Apr 10;5(4):e1000345. doi: 10.1371/journal.pcbi.1000345 (PMC2661023; doi:10.1371/journal.pcbi.1000345)
Supplement: Table S1 — Model comparison of cost functions (0.06 MB DOC) [file pcbi.1000345.s001.doc]

# Supplementary materials

Table S1: Model comparison of cost functions

| Model terms | Residual sum of squares | Cross-validation sum | AIC | Log marginal likelihood |
| --- | --- | --- | --- | --- |
| *λ* | 0.51 | 0.51 | -113.6 | 57.5 |
| *μ* | 1.74 | 1.74 | -40.2 | 20.7 |
| *ν* | 1.49 | 1.49 | -49.4 | 25.4 |
| *λ, μ* | 0.27 | 0.28 | -149.9 | 76.2 |
| *λ, ν* | 0.31 | 0.32 | -141.1 | 71.8 |
| *μ, ν* | 1.34 | 1.38 | -54.0 | 28.4 |
| *λ, ν, μ* | 0.23 | 0.25 | -157.1 | 80.5 |

Models include terms for *λ*: effort cost, *μ*: normalized effort cost, *ν*: variability cost. Residual sum of squares is for the best fit of the model to the data. Cross-validation sums of squares is computed as the sum of squared errors over all observation, when fitting the model to the whole data set except that observation. In models with one cost-function term (no free parameter), the cross-validation sum of squares is equal to the residual sum of squares. The Akaike Information Criterion (AIC) is computed as 2 (number of free parameters) – 2 log (maximal likelihood) of the model. Low values indicate a superior model. The log marginal likelihood is computed through MCMC sampling of the posterior distribution. The last three terms correct all for the number of free parameters and show a superiority of the full model.
